# The Link between Health Complaints and Wind Turbines: Support for the Nocebo Expectations Hypothesis

**DOI:** 10.3389/fpubh.2014.00220

**Published:** 2014-11-11

**Authors:** Fiona Crichton, Simon Chapman, Tim Cundy, Keith J. Petrie

**Affiliations:** ^1^Department of Psychological Medicine, University of Auckland, Auckland, New Zealand; ^2^School of Public Health, University of Sydney, Sydney, NSW, Australia; ^3^Department of Medicine, University of Auckland, Auckland, New Zealand

**Keywords:** wind farms, infrasound, nocebo effect, psychological expectations, health scares, symptom reporting, environmental risks, media warnings

## Abstract

The worldwide expansion of wind energy has met with opposition based on concerns that the infrasound generated by wind turbines causes health problems in nearby residents. In this paper, we argue that health complaints are more likely to be explained by the nocebo response, whereby adverse effects are generated by negative expectations. When individuals expect a feature of their environment or medical treatment to produce illness or symptoms, then this may start a process where the individual looks for symptoms or signs of illness to confirm these negative expectations. As physical symptoms are common in healthy people, there is considerable scope for people to match symptoms with their negative expectations. To support this hypothesis, we draw an evidence from experimental studies that show that, during exposure to wind farm sound, expectations about infrasound can influence symptoms and mood in both positive and negative directions, depending on how expectations are framed. We also consider epidemiological work showing that health complaints have primarily been located in areas that have received the most negative publicity about the harmful effects of turbines. The social aspect of symptom complaints in a community is also discussed as an important process in increasing symptom reports. Media stories, publicity, or social discourse about the reported health effects of wind turbines are likely to trigger reports of similar symptoms, regardless of exposure. Finally, we present evidence to show that the same pattern of health complaints following negative information about wind turbines has also been found in other types of environmental concerns and scares.

## Introduction

In recent years, challenges to new wind farm developments have been mounted on the basis that exposure to sound, and particularly infrasound, generated by wind turbines poses a health risk (1). Unfortunately, addressing concerns about health effects has been complicated by a lack of clarity about what might be causing the symptoms reported. Perceived adverse health effects said to be experienced by people living near wind turbines include symptoms such as sleep disturbance, headache, earache, tinnitus, nausea, dizziness, heart palpitations, vibrations within the body, aching joints, blurred vision, upset stomach, and short-term memory problems (2). In this article, we explore factors that might explain symptom reporting attributed to wind farms and put forward the case for the nocebo expectations hypothesis; that symptom reporting can be explained by negative expectations, rather than any pathophysiological link between symptoms and wind farm sound. Research consistently indicates that the expectation of adverse health effects can itself produce negative health outcomes, which is a phenomenon known as the nocebo effect (3). Negative expectations generating nocebo responses have been shown to have a powerful influence on health outcomes in clinical populations (4), and reported symptom experiences in community samples (5).

## The Link between Wind Farm Sound and Health Complaints

When investigating the cause of symptom reporting attributed to any purported environmental hazard, it is axiomatic that the existence of a biological basis for symptomatic experiences is thoroughly explored, so that an organic cause of symptoms is not erroneously discounted (6). Given that symptom reporting has been attributed to wind farm sound (2), it is necessary to consider the evidence for any direct relationship between exposure to such sound and symptom reporting. Given reductions in mechanical noise, as a result of refinements to wind turbine design, aerodynamic sound is now the dominant source of noise from modern wind farms (7). This aerodynamic noise, which is generated as a result of the flow of air past the turbine blades, is present across a range of frequencies, from the audible to sub-audible infrasound (8).

At this time, studies have not found a direct causal link between living in the vicinity of wind farms, audible wind farm sound exposure, and physiological health effects (1). Audible sound levels, assessed at the nearest residence, have been consistently found to fall within accepted health and safety limits for ambient background noise, and evidence does not support a direct link between such sound exposure and symptom reporting (9). To elaborate further, although a small proportion of people report being annoyed by wind farm sound, particularly by detectable fluctuations of sound in the mid-frequency range (500–1000 Hz), the evidence does not indicate that exposure to such sound is directly causing adverse physiological effects in those living in the vicinity of wind farms (8). In addition, despite concerns that audible low frequency noise (20–200 Hz) produced by wind turbines is triggering symptomatic experiences, this is not supported by the scientific evidence (10).

Further, the evidence does not substantiate conjecture that exposure to sub-audible wind farm generated infrasound (sound below 20 Hz) is responsible for health complaints. It is important to note that exposure to infrasound is an everyday experience. Infrasound is constantly present in the external environment, caused by phenomena such as weather variations, air turbulence, ocean waves, traffic, and other machinery (11). Notably, the body and vestibular systems have evolved to prevent disturbance from infrasound generated from internal processes, such as respiration and heart rate, which is produced at higher levels than infrasound generated by wind farms (12). While sound in the infrasonic range may become audible at sufficiently high pressure levels, infrasound produced by wind turbines is below the threshold of human perception (11, 13), and research does not support the existence of adverse health effects of exposure to infrasound at sub-audible levels (14). Importantly, a recent investigation found the contribution of wind turbines to measured infrasound levels at residential locations near wind farms was insignificant in comparison with the background level of infrasound in the environment (15). Given consistent evidence that infrasound produced by wind turbines does not exceed typical levels of infrasound found in everyday urban or rural environments, health impacts of infrasound produced by wind turbines are not indicated (12, 16).

As the evidence does not support a direct link between audible or sub-audible sound generated by wind turbines and reported symptomatic experiences by people living in the vicinity of wind farms, it is apparent that factors beyond exposure to wind turbine sound are implicated in symptom reporting.

## Perception of Health Risk and Expectations

There is accruing evidence that some people facing the prospect of a new wind farm near their residence, or currently living within the vicinity of a wind farm, are genuinely fearful of the potential health effects of operating wind turbines (1). This has relevance as evidence shows a relationship between assessment of health risk and symptom reporting, which does not depend upon whether a health risk is genuine (17). This is seen in community examples where there has been an error about exposure to a perceived toxic agent. In one such case, symptom complaints attributed to exposure to electromagnetic radiation from a mobile phone tower occurred when the tower itself was not yet active (18).

In fact, extreme increases in symptom reports, in instances of both genuine and perceived toxic exposure to harmful agents, have been repeatedly shown in community settings (19) with strength of environmental concern being a critical factor in predicting the occurrence of symptom complaints (20). This was highlighted in a study in which participants, from 10 villages in Germany, had their sleep monitored over 12 nights during which they were exposed to sham signals and electromagnetic field signals from an experimental base station (21). There was no evidence for short-term physiological effects of electromagnetic fields emitted by mobile phone base stations on sleep quality, but findings demonstrated a negative influence on objective and subjective sleep quality in subjects who were concerned that proximity to mobile phone base stations might negatively affect health.

Evidence shows that health-related worries about perceived environmental hazards inform negative expectations, which in turn draw attention to body processes and shape how individuals decipher symptoms [e.g., Ref. (22)]. Negative expectations translate into symptomatic experiences, because focused attention to the body has the tendency to draw awareness to common sensations that might otherwise go unnoticed (23). Further, increased anxiety itself causes a rise in physiological activity giving rise to symptoms such as dry mouth and rapid heart-beat (23). Evidence suggests people may misinterpret symptoms of hypervigilance and anxiety as a sign of illness, particularly if symptoms experienced are consistent with concerns about health (24).

Recently, there has been a noticeable rise in the number of people expressing concern about health effects presented by the sound generated by wind farms, and fears about health risk have emerged as a key predictor of opposition to wind farm development (25, 26). Such fears are more prominent in countries where wind farms are relative new comers on the landscape, which aligns with consistent evidence of associations between the introduction of new technologies, community concern about related health risks, and symptom reporting (27, 28).

## Matter of Expectation

While the operation of modern commercial wind farms commenced more than 20 years ago in several nations, widespread claims that exposure to wind farm sound produces adverse, often acute and immediate, symptomatic experiences, are much more recent (29). This change is reflected in the shifting focus of community opposition to wind farms over time. Historically, community opposition to wind farms has centered on concerns about depreciation of property values, problems with esthetic integration on the landscape, and apprehension about the intrusiveness of noise produced by wind turbines (30, 31). However, in recent years, concern about the adverse health risk of exposure to wind turbine sound has repeatedly emerged as a new focal point of community opposition to wind farms, indicating a change in the way in which wind farms are now perceived (1).

Such concern, as well as a dramatic amplification of symptom reports (29), coincided with the promotion in 2009 of the self-published book *Wind Turbine Syndrome-A Natural Experiment* (2), also available and summarized on the internet. The book portrays infrasound produced by wind turbines as a threat to health, and explicitly sets out the physical symptoms and health effects to be expected by those living in proximity to a wind farm. Given that wind farms simultaneously generate infrasound and audible sound, negative health information about infrasound is likely to influence the perception of wind farm sound in its entirety. Further, although the narrative of the book emphasizes the perniciousness of the sub-audible components of wind farm sound, it also sets out health concerns about audible sound, particularly low frequency audible wind farm sound. Thus, health concerns triggered by the type of information contained in the book are likely to inform negative expectations extending to both the audible and sub-audible components of wind farm sound exposure.

The concurrence of the publication of *Wind Turbine Syndrome-A Natural Experiment* and an increase in symptom reporting attributed to wind farms (29) supports the argument that symptoms are more likely due to negative expectations triggered by health information, rather than being caused by pathogenic exposure to wind farm sound. This is exemplified in a study assessing historical complaints, in relation to 51 Australian wind farms operating from 1993 to 2012 (29). Findings illustrated that, prior to 2009, health and noise complaints were rare, despite small and large wind farms having operated in Australia for many years. The study found that 90% of complainants made their first complaint post 2009, after anti-wind farm campaigners disseminated information about the purported health effects of wind farms. Further, the majority of complaints were confined to the six wind farms targeted by anti-wind farm campaigners, indicating complainants had accessed negative health information (29).

Additional support for the involvement of negative expectations, in relation to the increase in symptom reporting seen since 2009, is also provided by recent field research demonstrating that people higher in negative-oriented personality traits are more likely to report higher levels of perceived noise (unrelated to actual noise levels) and more non-specific physical symptoms around wind farms (32). Experimental research demonstrates that individuals with higher levels of negative affect are more susceptible to the influence of expectations about health effects created by suggestion and more likely to report expectation consistent symptoms (33).

The ascription of a disease label “Wind Turbine Syndrome” is a powerful way to create health concerns and set expectations. Where individuals adopt disease labels to reflect symptomatic experiences attributed to environmental causes they are more likely to be concerned about the environmental health risk posed, and less likely to be reassured by scientific investigation if it indicates there is no link between the perceived environmental hazard and symptoms (34). The use of an illness label “Wind Turbine Syndrome” (2), along with a widely publicized and explicated list of syndrome symptoms, not only creates the impression that there is a risk that those living near wind turbines will develop a recognized medical condition, but also creates a comprehensive idea of expected symptoms. Simply reading about symptoms of an illness can prompt self-detection of disease specific symptoms, a phenomenon seen in medical student disease. Here, medical students, in the course of learning about an illness, start to experience symptoms indicative of the disease studied (35, 36). The process of learning about an illness appears to generate a cognitive representation of the illness, or mental schema, which guides the way in which internal sensory information is attended to, so that symptoms or sensations that align with the schema are noticed and reported. Symptoms that are inconsistent with the schematic representation of the relevant illness are likely to be overlooked or discounted (37).

Thus, negative expectations operate as a blueprint or heuristic for the type of symptoms attended to and reported. In a clinical research setting, a substantial number of patients, randomized to the placebo arms of placebo controlled drug trials, experience and report symptoms reflective of the side effects of active treatment [e.g., Ref. (38)]. In an experimental study, participants inhaling a benign substance, described to them as a “suspected environmental toxin” known to cause headache, nausea, itchy skin, and drowsiness, reported increases in symptoms, particularly in relation to symptoms they had been told they might expect to experience (39).

Therefore, merely being aware of the type of symptoms that have been attributed to wind turbines is likely to trigger an expectancy directed cognitive body search, whereby the body is selectively monitored for sensations and symptoms consistent with ideas about the physiological effects of exposure to wind farms. During this process, individuals will be inclined to notice common symptoms, which align with expectations and to interpret ambiguous sensations in accordance with such beliefs (40). This was demonstrated in a double-blind provocation study, where participants who watched material from the internet suggesting that infrasound produced by wind farms generated symptoms, reported significant increases from pre-exposure assessment, in the number and intensity of symptoms experienced during exposure to both infrasound and sham infrasound (41). Importantly, elevations in symptom reporting, during exposure periods, coincided with information about the precise symptom profile, said to be related to infrasound exposure. During both exposure periods, participants reported more symptoms characterized as typical symptoms of infrasound exposure, than symptoms differentiated as atypical symptoms of exposure to infrasound. Results suggested that expectations formed by accessing negative health information about wind farm sound could be providing a pathway for symptom reporting in community settings.

## Expectations and Misattribution

It is important to note that many of the symptoms said to arise from exposure to wind farms, such as headache, fatigue, concentration difficulties, insomnia, gastrointestinal problems, and musculoskeletal pain, are commonly experienced by healthy individuals (23). If people are worried about the health effects of an environmental agent and form symptom expectations, they are also more likely to notice and misattribute their current symptomatic experience to that environmental agent. This can occur even when symptoms are more consistent with everyday experiences and may, under different circumstances, be explained as just part and parcel of normal life (22). Given that the symptoms said to be associated with wind turbines, such as tinnitus, sleep problems, and headache, are extremely common in the general community (42–44), many hearing about a putative connection with wind turbine exposure may be persuaded that health problems they experience can be attributed to this exposure. An analysis of symptom reporting by people living in the vicinity of wind turbines in Canada indicated that the prevalence of reported symptoms was consistent with symptom prevalence in the general population, suggesting that people are likely to be misattributing their ordinary experience of common symptoms to wind turbines, rather than becoming more symptomatic (45).

Many of the symptoms associated with wind turbines, such as dizziness and heart palpitations, are also stress-related concomitants of autonomic arousal associated with anxiety and distress (46). Further, evidence indicates a bidirectional relationship between anxiety and insomnia (47), so that people who are anxious about the health effects of wind farms may experience sleep difficulties because of this anxiety, and sleep difficulties may, in turn, exacerbate the experience of physiological symptoms of anxiety. These symptoms may then be misattributed to wind farm sound, if there is an expectation that wind farm sound poses a health risk.

Evidence also indicates that fears associated with beliefs that innocuous stimuli have dangerous health consequences, engenders associations between such stimuli and stress-related symptoms, so that exposure to such stimuli may become a cue for symptom expression (48). Therefore, detecting wind turbine noise may facilitate symptom expression because, for those concerned about the health effects of wind turbines, hearing the noise signifies exposure to a perceived environmental hazard. Such an interpretation would provoke anxiety, resulting in heightened physiological arousal and stress-related symptoms.

Interestingly, evidence suggests that individuals are much less likely to be annoyed by wind turbine noise if they unable to see wind turbines from their dwelling, even if the sound itself is at a relatively high level (49). Where individuals are worried about the health effects of wind turbines, the visibility of wind turbines from a residence is likely to be a particularly concrete reminder of their concern, thus perpetuating anxiety and related physiological arousal. Therefore, both audibility of sound and visibility of a wind turbine may act as situational cues for symptom expression, triggering stress-related symptoms, thereby reinforcing health concerns (48).

Concerns about a perceived environmental hazard and corresponding negative expectations can also lead to misattribution of current illness, so that illnesses are viewed as a reaction to environmental exposure rather than the result of aging or other disease processes. Over the past 50 years, an increasing concern about the environment appears to have led to heightened sensitivities to environmental change, which have also impacted on the way people perceive illness and disease (17). Individuals are more inclined than previous generations to view ill health as a by-product of a toxic environment, and to worry about the enduring health effects of environmental changes. The propensity to look for external environmental causes for ill health is illustrated by research indicating a tendency among cancer survivors of the 10 most common cancers to believe environmental factors play a much more significant role in carcinogenesis than scientific evidence warrants (50). Therefore, an environmental change, particularly involving the use of an emerging technology, is likely to be regarded with suspicion and trigger expectations impacting on the way individuals interpret their own symptomatic experiences. Diseases such as diabetes, skin cancer, and stroke, with much more established etiology, have instead been ascribed to wind farms indicating a process of misattribution (51).

## Media Health Warnings and Expectations

A recent study has demonstrated that the upsurge in noise and health complaints seen in Australia since 2009 has arisen primarily in localities where there has been targeted publicity about the alleged harmful impacts of wind farms (29). Two entire Australian states with wind farms, but no history of anti-wind farm advocacy, had no reported instances of health or noise complaints. Findings are consistent with research indicating that media warnings about potential harm from environmental factors may create health concerns prompting symptom reporting, even in the absence of objective health risk (48). Merely watching a television report about the supposed adverse effects of Wifi has been shown to elevate concern about the health effects of electromagnetic fields and increase the likelihood of experiencing symptoms following exposure to a sham Wifi signal (52).

In the case of wind farms, recent media stories have been shown to contain fright factors likely to trigger fear, concern, and anxiety about the health risk posed by wind turbines (53). Assertions about the adverse impacts of wind farm sound have been widely disseminated by the media, particularly via anti-wind farm internet websites, and have led to misconceptions about infrasound generated by wind turbines and a conviction in some that wind farms cause a myriad of health complaints (12) Conjecture about the adverse health effects of wind farms is a consistent theme in public discourse about wind turbines found in media reports embodied in headlines such as *“Wind turbines cause heart problems, headaches and nausea*…*“* (54); *“Coming to a house, farm, or school near you? Wind Turbine Syndrome*… “ (55); and television news items such as “*Wind Turbines cause health problems, residents say*” (56). Further, misleading reports about the impact of living in the vicinity of wind farms, such as inaccurate accounts of home abandonment and emotive references to wind farm refugees, is also liable to create disquiet (57).

It has been verified in a recent double-blind provocation study that the kind of information disseminated in the case of wind farms elevates health concerns and creates corresponding negative expectations, which result in symptomatic experiences. Participants viewing a DVD, containing extracts from the internet outlining the alleged health effects of infrasound generated by wind turbines, reported increased concern about the health effects of sound produced by wind farms, which was associated with amplification of symptom reporting during both genuine and sham exposure to infrasound (41). Results showed negative expectations may be created by media portrayal of alleged health risks posed by the sound created by wind turbines, which could explain symptom reporting around wind farms.

The profound effect of the media narrative on the experience of wind farm sound was confirmed in a follow-up study in which subjective health was influenced in either positive or negative directions, depending on how the sound was portrayed. In keeping with previous findings, participants with negative expectations, formed from media warnings about infrasound, reported increased symptoms and deterioration in mood during simultaneous exposure to infrasound and audible wind farm sound (58). In contrast, participants delivered positive expectations derived from information extracted from the internet about the alleged therapeutic effects of infrasound, experienced an improvement in symptomatic experiences and mood. Findings demonstrated the malleability of symptomatic responses and the power of information disseminated through the media to create expectations, which determine how wind farm sound is experienced. It was particularly telling that positive expectations about infrasound triggered a placebo response in participants listening to audible wind farm sound, while being exposed to infrasound. This highlights that exposure to audible wind farm sound can be a pleasurable experience, if the narrative about the sound is depicted positively. The study provides encouraging indications that if information disseminated about wind farm sound is framed in more neutral or benign ways, then reported symptoms or negative health effects can be ameliorated.

## Expectations Created by Social Interactions

It is important to bear in mind that the experience of symptoms attributed to wind turbines occurs in community settings, and in a social context where there are a range of opinions, concerns, and pressure group activity about the construction of wind farms and about possible health risks associated with them (1, 30). Evidence has shown residents’ fears about the health effects of wind turbines are increasingly becoming the focal point of community public consultation meetings, formed as part of resource consent and environmental assessment processes that relate to wind farms (1). Expectations can be learned from such social interactions (59), and may also be created and reinforced by observation and modeling (Faasse et al. under review). The potential effect of observation on symptom experience is indicated in an experimental study demonstrating that one-third of healthy controls, when exposed to images of other people in pain, reported pain in the same location as the observed pain (60). Further, in an experimental study in which participants inhaled an inert substance portrayed as a possible environmental toxin, seeing someone exhibiting expected symptoms increased participant reports of those specific symptoms, illustrating the phenomenon of contagion by observation, seen in mass psychogenic illness (61).

There are various avenues for observation and modeling of symptoms within communities where wind farms are established. Neighbors and members of the wider community may be exhibiting and talking about their symptomatic experiences, which they attribute to wind farms. Television reports about the health effects of wind turbines have also incorporated interviews with symptomatic people, describing their experiences in detail, providing another medium by which symptoms may be modeled [e.g., Ref. (56)]. These interviews can usually be accessed on the internet, so people researching the effects of wind farms can observe modeled behavior with ease.

There are also indications that, where symptoms are attributed to wind turbines, health problems are reported by everyone within the affected household, including children [e.g., Ref. (2)]. This suggests that familial modeling may play a role in symptom reporting, particularly in relation to affected children. Parental pain and symptom modeling is implicated in the development of unexplained pain and somatic complaints in pediatric populations (62, 63).

## Annoyance and Expectations

It seems apparent that elevated concern about the health effects of living in the vicinity of wind farms, and the related formation of negative expectations, is also exacerbating reported annoyance with wind farm sound. There is much variability between studies in relation to the extent of reported wind farm noise annoyance indicating that contextual matters are influencing annoyance reactions. Related studies undertaken in Sweden and the Netherlands have indicated that approximately 10–20% of residents living in proximity to wind farms find wind turbine noise annoying, and 6% of residents find wind turbine noise very annoying, at 35–40 dB exposure (7, 49, 64). However, another study conducted in New Zealand reported that 59% of respondents living within 2 km of a wind farm experienced noise annoyance (65). The New Zealand study was undertaken at a time when there had been adverse publicity about expected noise and health effects of living in the vicinity of the wind farm in question, including a story that aired on free to air television (66). Understanding the factors that contribute to annoyance is important because, although noise annoyance is not in itself a disease or health state, annoyance is related to distress, which can lead to the experience of stress-related symptoms (9, 67).

Being annoyed by noise is related to a range of personal and situational variables, beyond the acoustic characteristics of noise (68, 69), and psychosocial factors account for more variation in individual annoyance, than objective measures of noise level (70). Experimental work indicates that not being aware of the source of sound is associated with reduced noise annoyance in people exposed to wind farm sound, further confirming that the context of sound exposure has more relevance for annoyance assessment, than the acoustic properties of wind farm sound (71). Importantly, a strong relationship has been found between concern about the negative health effects of noise and noise annoyance (72). The evidence also shows that wind turbine noise annoyance is more strongly related to other negative attitudes about wind turbines, particularly the visual impact of wind turbines on the land scape, than to sound level (7, 49). Thus, rhetoric that creates health concerns about wind turbine sound, and presents a negative view of wind farms, is likely to influence not just symptom reporting and distress, but reported noise annoyance.

There is compelling evidence that creating a positive context for the experience of wind farm sound, has a correspondingly positive impact on reported annoyance. A field study conducted in The Netherlands indicated that respondents who benefited economically from wind turbines, by either full or partial turbine ownership or by receipt of other economic benefits, such as a yearly income, were less annoyed by wind turbine noise than other respondents, despite exposure to higher sound levels (49). Notably, there were no differences in either likelihood to notice sound, or subjective noise sensitivity between those who did or did not derive economic benefit. However, there were attitudinal differences. Respondents who benefited economically were less negative both about wind turbines in general, and about the visual impact of wind turbines on the landscape. Results suggest that experiencing wind farm sound in a positive context decreases the likelihood of forming negative views of wind turbines associated with annoyance. This provides promising indications that changing the narrative around wind farms, so that worried residents become less concerned about their proximity to wind farms and adopt more positive expectations and attitudes, might not only alleviate symptom reporting but also reduce noise annoyance.

## Patterns of Health Complaints Seen in Other Instances of Perceived Toxic Exposure

It is relevant to note that symptom reporting, in response to perceived exposure to a toxic agent when no plausible health threat is posed, has been seen throughout history (17). Francis Bacon (1561–1626) noted “*infections*…*if you fear them, you call then upon you*” (73). In one pertinent example, a dramatic elevation in reported symptoms in a community setting in Memphis followed a health scare fueled by media messages that the town was located in close proximity to an old toxic waste dump (74). While a comprehensive examination of soil toxicity revealed no hazard was presented, health fears did not abate until it became apparent authorities were mistaken as to the locality of the dump, which had actually been situated many miles from the town (19). Although symptom reporting then subsided, some residents continued to insist they experienced symptoms from the phantom dump site.

Further, the advent of new technologies has consistently been associated with the development of subjective illness complaints, involving a constellation of symptoms, akin to those attributed to wind farms (28, 75). For instance, in 1889, following the increasing use of the telephone, The British Medical Journal cautioned about the emergence of *“telephone tinnitus”* in respect of which “*the patients suffered from nervous excitability, with buzzing noises in the ear, giddiness, and neuralgic pains”* (76). With striking parallels, almost a century later, the experience of a range of non-specific symptoms such as headache, fatigue, tinnitus, and concentration problems have been attributed by some individuals to exposure to electromagnetic fields via mobile telephones (77). This occurs despite the fact there is no generally accepted causal bio-electromagnetic mechanism, by which such symptoms would be triggered (78). Given that provocation studies have repeatedly shown that sham electromagnetic exposure is sufficient to activate symptoms in individuals who believe they are sensitive to electromagnetic fields, the evidence suggests the involvement of nocebo responses; that it is anxiety about exposure and related negative expectations, which are triggering symptomatic experiences (52).

## Conclusion

An analysis of the evidence concerning symptom reporting attributed to sound produced by wind farms supports the nocebo expectation hypothesis; that health complaints can be explained by the influence of negative expectations. It is apparent that symptom reporting coincided with an increase in health concern about wind farms promoted by a book and internet sites focused on highlighting the purported heath dangers posed by sound, particularly infrasound produced by wind turbines. Such information, which has been further circulated though social discourse and media reporting, is liable to trigger health concerns and related symptoms of anxiety, while also creating a blueprint for what symptoms can be expected – expectations, which, in turn, are likely to guide the type of symptoms noticed and reported. This is supported by epidemiological evidence that increased symptom reporting has occurred in locations where there has been targeted dissemination of negative health information about wind farms, indicating that exposure to such information is shaping symptomatic experiences. Experimental work also suggests that it is expectation rather than wind farm sound exposure that is responsible for symptom complaints.

Symptom reporting is also consistent with patterns of health complaints seen in other environmental health scares involving benign exposure, and which often follow the introduction of new technologies. Importantly, indications that negative expectations are implicated in symptomatic experiences ascribed to wind farms aligns with evidence that instances of symptom reporting attributed to perceived environmental hazards and exposure to modern technologies have been triggered by nocebo responses.

Understanding the underlying cause of health concerns and symptom complaints, which have arisen in communities in which wind farms have been proposed and developed, is critical if such concerns are to be addressed, and symptom reporting alleviated. Given indications of the determinative role of negative expectations in creating and maintaining symptom reporting, successful strategies to address health complaints are likely to involve changing the narrative about wind farms, to create more positive expectations.

## Author Contributions

All authors contributed to the conceptualization, writing, and editing of this manuscript.

## Conflict of Interest Statement

There are no commercial, financial, or other competing factors, which could be viewed as constituting a conflict of interest influencing the article’s content. The authors have not received any funding for the article, which is substantially based on the authors’ own epidemiological and experimental research. The authors are independent, academic professionals working in the area of psychological medicine, public health, and medicine, with a shared expertise about psychological factors, which impact on symptom reporting in response to perceived environmental risks. To this end, Simon Chapman has previously provided expert advice on psychogenic aspects of complaints about wind farm to lawyers acting for Infigen. Further, Keith J. Petrie has previously provided expert evidence for the NZ Environment Court and the Canadian Environment Review Tribunal on psychological aspects of complaints about wind farm developments.
